# Disentangling the relationship between cholesterol, aggression, and impulsivity in severe mental disorders

**DOI:** 10.1002/brb3.1751

**Published:** 2020-07-17

**Authors:** Gabriela Hjell, Lynn Mørch‐Johnsen, René Holst, Natalia Tesli, Christina Bell, Synve Hoffart Lunding, Linn Rødevand, Maren Caroline Frogner Werner, Ingrid Melle, Ole Andreas Andreassen, Trine Vik Lagerberg, Nils Eiel Steen, Unn Kristin Haukvik

**Affiliations:** ^1^ NORMENT Division of Mental Health and Addiction Oslo University Hospital & Institute of Clinical Medicine University of Oslo Oslo Norway; ^2^ Departments of Psychiatry and Clinical Research Ostfold Hospital Gralum Norway; ^3^ Department of Biostatistics Institute of Basic Medical Sciences University of Oslo Oslo Norway; ^4^ Department of Adult Psychiatry Institute of Clinical Medicine University of Oslo Oslo Norway; ^5^ Centre of Research and Education in Forensic Psychiatry Oslo University Hospital Oslo Norway

**Keywords:** aggression, bipolar disorder, cardiovascular disease, cholesterol, schizophrenia

## Abstract

**Objective:**

Low total cholesterol has been linked with adverse mental symptoms such as aggression and impulsivity in severe mental disorders (SMDs). This putative association may affect the clinician's decision making about cholesterol lowering in this patient group. Here, we investigated the associations between cholesterol levels, aggression, and impulsivity in a large representative sample of in‐ and outpatients with SMD.

**Methods:**

Patients with schizophrenia‐ or bipolar spectrum disorders (*N* = 1 001) underwent thorough clinical characterization and blood sampling (total cholesterol, low‐density lipoprotein cholesterol, high‐density lipoprotein cholesterol). Aggression was characterized by the Positive and Negative Syndrome Scale Excited Component. Impulsivity was measured with the Barratt Impulsiveness Scale in a subsample of patients (*N* = 288). We used a multinomial logistic regression model to analyze the association between cholesterol and aggression and a multiple linear regression model to analyze the association between cholesterol and impulsivity, while controlling for confounders.

**Results:**

We found no significant associations between cholesterol levels and aggression or impulsivity. There were no significant interactions between cholesterol and diagnostic group or inpatient versus outpatient status. Controlling for medication use, body mass index, alcohol or illicit substance use did not affect the results.

**Conclusion:**

In this large sample of patients with schizophrenia‐ and bipolar spectrum disorders, we found no associations between cholesterol levels and aggression or impulsivity. This has clinical implications as patients with SMD are at increased CVD risk and currently undertreated with statins.

## Graphical abstract

In context of the urgent need to improve cardiovascular health of patients with severe mental disorders, we investigated the associations between cholesterol levels and aggression in a sample of in‐ and outpatients with schizophrenia‐ and bipolar spectrum disorders. This large representative sample of patients with severe mental disorders revealed no significant associations between cholesterol levels and aggression or impulsivity.

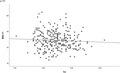

## 1 SIGNIFICANT OUTCOMES

- A large representative sample of patients with schizophrenia‐ and bipolar spectrum disorders revealed no significant associations between cholesterol levels and aggression or impulsivity.

## 2 LIMITATIONS

- Despite the comprehensive range of variables accounted for in our analysis, the process of controlling for potential confounders was limited by the observational design.
- The highest levels of aggression were scarcely represented among the study participants.

## 3 INTRODUCTION

Patients with severe mental disorder (SMD) such as schizophrenia and bipolar disorder are at increased risk of adverse outcomes including aggressive behavior, suicide, and premature mortality (Fazel et al., 2015; Fazel, Wolf, Palm, & Lichtenstein, 2014; Fleischman, Werbeloff, Yoffe, Davidson, & Weiser, 2014; Hayes, Miles, Walters, King, & Osborn, 2015; Walter et al., 2019). Cardiovascular diseases (CVDs) are among the leading causes of death in patients with SMD (Olfson, Gerhard, Huang, Crystal, & Stroup, 2015; Swaraj et al., 2019; Tanskanen, Tiihonen, & Taipale, 2018). Cholesterol lowering has strong protective effect on CVD mortality (Benjamin et al., 2018; Boekholdt et al., 2014), and the current cholesterol management guidelines (Grundy et al., 2019; Piepoli et al., 2016) advise initiation of statin therapy based on calculations of CVD risk. As a step toward personalized cholesterol management, the excessive CVD risk in SMDs (Laursen et al., (2013)) has been taken into account in the development of CVD risk assessment tools (Hippisley‐Cox, Coupland, & Brindle, 2017; Osborn et al., 2015). Low total cholesterol (TC) has, however, been linked with adverse mental symptoms such as aggression and impulsivity in SMDs (Tomson‐Johanson and Harro, (2018)). As such, the recommendations regarding cholesterol management may risk potentiating aggressive dispositions. Although most patients with SMD will never be aggressive, aggression and violent acts challenge everyday psychiatric clinical practice. Establishing the association between cholesterol and aggression and impulsivity in patients with SMD is therefore of high clinical relevance for personalized CVD prevention.

Psychological effects of cholesterol lowering have been robustly addressed in the general population (Collins et al., 2016). Even though an early meta‐analysis of primary CVD prevention randomized controlled trials (RCTs) linked decrease in TC to an increase in violent deaths caused by accident, homicide, and suicide (Muldoon, Manuck, & Matthews, 1990), subsequent research did not show adverse psychological effects of cholesterol lowering in the general public (Golomb et al., 2015; Muldoon, Manuck, Mendelsohn, Kaplan, & Belle, 2001). An explicit or implicit exclusion of individuals with SMD in these RCTs may limit our knowledge about psychological effects of cholesterol lowering in SMD patient group (Rothwell, 2005). Moreover, individuals with SMD might be particularly susceptible to potential adverse psychological effects of low TC. Genome‐wide association studies implicate a role of cholesterol metabolism in SMDs (Andreassen et al., (2013)). Cholesterol has been shown to interact with serotonin (Kaplan et al., 1994; Vevera et al., 2016), which is central in the pathophysiology of mental disorders (Lucki, 1998) and aggression (Coccaro, Fanning, Phan, & Lee, 2015; Klasen et al., 2019). Despite the protective role of the blood–brain barrier against direct effects of circulating levels of cholesterol on the brain, indirect mechanisms mediated by cholesterol metabolites have been demonstrated (Olsson et al., 2017).

Cholesterol and adverse mental symptoms have been investigated over the past three decades in a number of smaller‐to‐medium scaled studies in psychiatric settings (Apter et al., 1999; De Berardis et al., 2013; Eriksen, Bjorkly, Lockertsen, Faerden, & Roaldset, 2017; Huang & Wu, 2000; Kavoor, Mitra, Kumar, Sisodia, & Jain, 2017; Mufti, Balon, & Arfken, 1998; Roaldset, Bakken, & Bjorkly, 2011; Steinert, Woelfle, & Gebhardt, 1999; Suneson et al., 2019; Troisi, 2011; Wu et al., 2016), often showing inverse associations between TC and degree of aggression, impulsivity, or suicidality. Moreover, low TC has been associated with more frequent acts of aggression in forensic psychiatric populations (Hillbrand, Spitz, & Foster, 1995; Paavola, Repo‐Tiihonen, & Tiihonen, 2002). A meta‐analysis of the relationship between cholesterol and suicidality has shown inverse association between TC and suicidality (Wu et al., 2016). Several studies have shown inverse associations between TC and aggression (Hillbrand et al., 1995; Mufti et al., 1998; Paavola et al., 2002; Roaldset et al., 2011; Suneson et al., 2019) or impulsivity (Kavoor et al., 2017; Troisi, 2011), whereas other studies have reported no significant associations between TC and aggression (Apter et al., 1999; Eriksen et al., 2017; Huang & Wu, 2000; Kavoor et al., 2017; Steinert et al., 1999) or impulsivity (Apter et al., 1999). Findings among those studies that have investigated high‐density lipoprotein cholesterol (HDL‐C) (Eriksen et al., 2017; Kavoor et al., 2017; Paavola et al., 2002; Suneson et al., 2019; Troisi, 2011) vary from positive (Paavola et al., (2002)), to inverse (Eriksen et al., (2017)), to nonsignificant (Troisi, (2011); Kavoor et al., 2017; Suneson et al., 2019) associations with aggression. Additionally, inverse associations between low‐density lipoprotein cholesterol (LDL‐C) and aggression (Paavola et al., 2002; Suneson et al., 2019) or impulsivity (Kavoor et al., 2017) have been reported. Taken together, the body of evidence appears to be split into suggestions of lipid profile with low cholesterol (TC, LDL‐C) and suggestions of no distinct lipid profile pattern related to aggression. These substantial inconsistencies in findings and a focus restricted to inpatient settings call for an investigation of the association between cholesterol and aggression in a large representative sample of patients with SMD.

The aim of the present study was to determine the associations between cholesterol levels and aggression and impulsivity by investigating a large sample of in‐ and outpatients with schizophrenia or bipolar disorder. We hypothesized that cholesterol levels (TC, LDL‐C) would be negatively associated with aggression and impulsivity in patients with SMD. For completeness, we aimed to provide insight into how HDL‐C contributes to the hypothesized TC associations. Hence, we postulated that HDL‐C also would be negatively associated with aggression and impulsivity in patients with SMD.

## 4 MATERIAL AND METHODS

### 4.1 Participants

The present study is part of the Thematically Organized Psychosis (TOP) study, which recruits patients with SMD from psychiatric in‐ and outpatient clinics of the major hospitals in Oslo, Norway (Rodevand et al., 2019). The main inclusion criterion was a diagnosis within the schizophrenia‐ or bipolar spectrum. Further inclusion criteria were age between 18 and 65 years, ability to give informed consent, and Norwegian language skills sufficient for valid assessments. Exclusion criteria comprised marked cognitive deficit (IQ scores below 70), neurological disorder, and history of severe head trauma. The study participants were recruited between the years 2002 and 2017. Patients (*N* = 1 001) were classified in the following diagnostic groups according to the Diagnostic and Statistical Manual of Mental Disorders Fourth Edition (DSM‐IV) (American Psychiatric Association, 1994): schizophrenia spectrum disorders (*N* = 601), including schizophrenia (*N* = 360), schizophreniform disorder (*N* = 31), schizoaffective disorder (*N* = 96), delusional disorder (*N* = 36), and psychotic disorder not otherwise specified (NOS) (*N* = 78); bipolar spectrum disorders (*N* = 400), including bipolar I disorder (*N* = 229), bipolar II disorder (*N* = 112), bipolar disorder NOS (*N* = 21), and major depressive disorder with psychotic features (*N* = 38).

### 4.2 Clinical measures

All participants were thoroughly assessed by trained psychologists and physicians. The diagnostic evaluation was based on the Structured Clinical Interview for DSM‐IV axis I disorders (SCID‐I) (First, Spitzer, Gibbon, & Williams, 1995), including a review of medical records. The inter‐rater diagnostic agreement has been estimated to a satisfying level of 82% with overall κ = 0.77 (95% CI: 0.60–0.94) (Rodevand et al., 2019). General psychiatric symptom load was evaluated using the Global Assessment of Functioning Split Version (GAF) (Pedersen, Hagtvet, & Karterud, 2007). Affective symptoms were assessed with the Young Mania Rating Scale (YMRS) (Young, Biggs, Ziegler, & Meyer, 1978) and the Calgary Depression Scale for Schizophrenia (CDSS) (Addington, Addington, & Schissel, 1990). The Positive and Negative Syndrome Scale (PANSS) (Kay, Fiszbein, & Opler, 1987) was used to rate current psychotic symptoms, achieving inter‐rater reliability of intraclass correlation coefficient at 0.82 (Rodevand et al., 2019). The PANSS consists of 30 items, each scored from 1 (no symptoms) to 7 (severe symptoms).

A measure of aggression among patients was obtained using the PANSS Excited Component (PANSS‐EC) (Montoya et al., 2011), which is a part of the Five Factor PANSS Model (White, Harvey, Opler, & Lindenmayer, 1997). The PANSS‐EC was calculated as a sum of the following items: P4‐excitement, P7‐hostility, G4‐tension, G8‐uncooperativeness, and G14‐poor impulse control (Lindenmayer et al., 2004). The PANSS‐EC is commonly used as a primary outcome measure in RCTs targeted at agitation and aggressive behavior in emergency psychiatric settings (Citrome, 2007; Zeller et al., 2017). Due to the distribution of PANSS‐EC scores in our sample, PANSS‐EC was grouped into the following categories reflecting the level of aggression: PANSS‐EC = 5 represents a group with no aggression symptoms (NAS), PANSS‐EC ranging from 6 to 10 represents a group with minimal level of aggression symptoms (MLAS), and PANSS‐EC > 10 represents a group with higher levels of aggression symptoms (HLAS). The unifactorial structure of the PANSS‐EC (Montoya et al., 2011) and PANSS rating criteria (Kay et al., 1987) were used as a background for the establishment of the cutoffs. Thus, the first group NAS was defined by the absence of all PANSS‐EC symptoms, the second group MLAS spanned from outer end of the normal range to suspected pathology, and PANSS‐EC scores indicative of more certain and expressed pathology were categorized into the third group HLAS. The PANSS assessment was conducted within one month of the blood sampling (median 9 days, interquartile range 9 days).

Impulsivity was measured with the Barratt Impulsiveness Scale (BIS‐11) (Patton, Stanford, & Barratt, 1995) questionnaire among a subgroup of patients (*N* = 288). The BIS‐11 consists of 30 items scored on a 4‐point Likert scale. Hence, the total score ranges from 30 to 120, with higher scores reflecting higher levels of impulsivity. Internal consistency of the BIS‐11 total score has been repeatedly reported as acceptable (Lindstrom, Wyller, Halvorsen, Hartberg, & Lundqvist, 2017; Patton et al., 1995; Reise, Moore, Sabb, Brown, & London, 2013; Stanford et al., 2009).

The Alcohol Use Disorders Identification Test (AUDIT) (Babor et al.,) and the Drug Use Disorders Identification Test (DUDIT) (Voluse et al., 2012) questionnaires were used to evaluate alcohol and illicit substance use. Information about psychopharmacological treatment was collected from medical records and by interview. Current dose in use relative to the defined daily dose (DDD) was calculated in line with the guidelines from the World Health Organization Collaborating Center for Drug Statistics Methodology (https://www.whocc.no/atc_ddd_index/). Body mass index (BMI) (kg/m^2^) was calculated based on weight and height measurements.

### 4.3 Serum analyses

Venous blood samples were collected in the morning after an overnight fast of at least 8 hr. Levels of TC, LDL‐C, and HDL‐C were measured at the Department of Medical Biochemistry, Oslo University Hospital, on routine instruments (Roche Diagnostics Cobas Integra 800, Roche Diagnostics Cobas 8000 e602/e801) using standard methods controlled by internal and external quality control samples. Until 2012, LDL‐C was calculated by the Friedewald formula (Martin et al., 2013), thereafter analyzed by an enzymatic colorimetric method.

### 4.4 Ethical considerations

The study was conducted in accordance with the Declaration of Helsinki. Written informed consent was obtained from all participants. The TOP study has approvals from the Regional Ethics Committee, the Norwegian Data Inspectorate, and the Norwegian Directorate of Health.

### 4.5 Statistical analyses

Statistical analyses were performed using IBM SPSS Statistics for Windows version 25.0 software package. Normality was assessed by Kolmogorov–Smirnov tests and Q‐Q plots. Differences in demographic and clinical characteristics across the aggression categories were investigated using chi‐squared tests, one‐way ANOVAs with post hoc *t* tests, or Kruskal–Wallis tests with post hoc Mann–Whitney U tests. All descriptive analyses were two‐tailed, with a significance level at 0.05.

We used a multinomial logistic regression model to analyze the association between cholesterol (TC, LDL‐C, HDL‐C) and the three levels of aggression (NAS, MLAS, HLAS), with cholesterol levels as the independent variable, adjusting for age, sex, and diagnostic group (schizophrenia‐ versus bipolar spectrum disorders). Then, we repeated the analyses with additional adjustments (BMI, inpatient versus outpatient status, alcohol use (AUDIT scores), illicit substance use (DUDIT scores) and use of psychotropic medication (dose relative to DDD) including antipsychotics, antidepressants, mood stabilizers, and lithium) in a subsample with all the covariates available (*N* = 689). Variation inflation factors (VIFs) were used to assess multicollinearity. Interactions between cholesterol levels (TC, LDL‐C, HDL‐C) and diagnostic group, inpatient versus outpatient status, and sex were tested. We applied the Bonferroni method to correct for multiple testing (three consecutive analyses with TC, LDL‐C, and HDL‐C as independent variable), with a significance level set at 0.017 (0.05/ 3).

The association between cholesterol (TC, LDL‐C, HDL‐C) and impulsivity (BIS‐11 total score) was assessed with a multiple linear regression model with impulsivity scores as the dependent variable, cholesterol levels as the independent variable and age, sex, and diagnostic group (schizophrenia‐ versus bipolar spectrum disorders) as covariates. Then, subanalyses (*N* = 259) with the additional covariates (BMI, inpatient versus outpatient status, alcohol use, illicit substance use, and use of psychotropic medication) were conducted. Multicollinearity was assessed using VIFs. Assumption of normality and presence of outliers were investigated by inspection of standardized residuals and Cook's distances. Interactions between cholesterol levels and diagnostic group, inpatient versus outpatient status, and sex were tested. We used the Bonferroni method to correct for multiple testing (analyses with TC, LDL‐C, and HDL‐C as independent variable), with a significance level at 0.017 (0.05/ 3).

## 5 RESULTS

### 5.1 Descriptive analyses

Demographic and clinical characteristics of the sample with aggression outcome are summarized in Table 1. There were significant differences in age (*p* < .001), education level (*p* < .001), and symptom load (GAF (*p* < .001), PANSS total score (*p* < .001), CDSS (*p* < .001), YMRS (*p* < .001) between the three aggression groups, with age decreasing and symptom load increasing gradually with increasing levels of aggression. Schizophrenia spectrum disorders were significantly more frequent than bipolar spectrum disorders in the HLAS group compared to the remaining groups (*p* = .009). There were no significant differences in uncorrected cholesterol levels across the three groups.

**Table 1:** Demographic and clinical characteristics of the sample with aggression outcome

	NAS (*N* = 270)	MLAS (*N* = 618)	HLAS (*N* = 113)	NAS versus MLAS	NAS versus HLAS	MLAS versus HLAS
	*N* (%)	*N* (%)	*N* (%)	*p*	*p*	*p*
Male	139 (51.5)	329 (53.2)	57 (50.4)	NS	NS	NS
Caucasian	221 (81.9)	511 (82.7)	85 (75.2)	NS	NS	NS
Schizophrenia spectrum disorder	151 (55.9)	368 (59.5)	82 (72.6)	NS	**.002**	**.009**
Bipolar spectrum disorder	119 (44.1)	250 (40.5)	31 (27.4)	NS	**.002**	**.009**
Outpatient^a^	160 (70.2)	381 (68.2)	64 (60.4)	NS	NS	NS
Currently smoking^a^	122 (46.4)	334 (55.2)	66 (59.5)	**0.017**	**.021**	NS
Use of statins	8 (2.9)	8 (1.3)	0 (0)	NS	NS	NS

	Mean (*SD*)	Mean (*SD*)	Mean (*SD*)	*p*	*p*	*p*
GAF‐S	54.4 (14.5)	48.3 (12.4)	38.9 (10.6)	**<.001**	**<.001**	**<.001**
GAF‐F^a^	52.8 (13.5)	48.7 (12.9)	40.3 (10.7)	**<.001**	**<.001**	**<.001**
PANSS total score^a^	45.6 (12.3)	57.3 (14.1)	73.5 (16.6)	**<.001**	**<.001**	**<.001**
TC in mmol/l	5.14 (1.07)	5.08 (1.07)	4.99 (0.98)	NS	NS	NS
LDL‐C in mmol/l	3.20 (0.97)	3.16 (0.95)	3.10 (0.90)	NS	NS	NS
HDL‐C in mmol/l	1.41 (0.41)	1.40 (0.42)	1.34 (0.44)	NS	NS	NS

	Median (IQR)	Median (IQR)	Median (IQR)	*p*	*p*	*p*
Age	30.0 (15)	29 (15)	25 (11)	**.016**	**.002**	**<.001**
Years of education	13 (3)	12 (3)	12 (2)	**<.001**	**<.001**	**<.001**
PANSS‐EC	5 (0)	7 (2)	12 (2)	**<.001**	**<.001**	**<.001**
CDSS^a^	3 (6)	5 (7)	7 (9)	**<.001**	**<.001**	**.001**
YMRS^a^	1 (4)	2 (8)	8 (9)	**< 0.001**	**<.001**	**<.001**
AUDIT^a^	5 (9)	4 (8)	6 (10)	NS	NS	NS
DUDIT^a^	0 (3)	0 (2)	0 (4)	NS	NS	NS
Antipsychotics^a^, ^b^	0.75 (1.23)	0.75 (1.50)	0.67 (2.00)	NS	NS	NS
Lithium^a^, ^b^	0 (0)	0 (0)	0 (0)	NS	**.017**	NS
Mood stabilizers^a^, ^b^	0 (0)	0 (0)	0 (0)	NS	NS	NS
Antidepressants^a^, ^b^	0 (0.27)	0 (1.00)	0 (0.47)	**.008**	NS	NS
BMI^a^	25.7 (5.6)	25.1 (5.8)	24.1 (6.7)	**.042**	**.028**	NS

Abbreviations: AUDIT, Alcohol Use Disorders Identification Test; BMI, body mass index; CDSS, Calgary Depression Scale for Schizophrenia; DUDIT, Drug Use Disorders Identification Test; GAF‐F, Global Assessment of Functioning Split Version‐Function; GAF‐S, Global Assessment of Functioning Split Version‐Symptoms; HDL‐C, high‐density lipoprotein cholesterol; HLAS group with higher levels of aggression symptoms; IQR, interquartile range; LDL‐C, low‐density lipoprotein cholesterol; MLAS, group with minimal level of aggression symptoms; NAS, group with no aggression symptoms; NS, nonsignificant; PANSS, Positive and Negative Syndrome Scale; PANSS‐EC, Positive and Negative Syndrome Scale Excited Component; *SD*, standard deviation; TC, total cholesterol; YMRS, Young Mania Rating Scale.

^a^ Information about inpatient versus outpatient status available for *N* = 893, about smoking status for *N* = 979, about GAF‐F for *N* = 999, about PANSS total score for *N* = 996, about CDSS for *N* = 697, about YMRS for *N* = 885, about AUDIT for *N* = 814, about DUDIT for *N* = 822, about antipsychotics for *N* = 982, about lithium *N* = 988, about mood stabilizers *N* = 988, about antidepressants *N* = 988, and about BMI for *N* = 964.

^b^ Psychotropic medications as current daily dose relative to defined daily dose.

*p* ≤ .05 in bold.

Demographic and clinical characteristics of the subsample with impulsivity outcome are summarized in Table 2.

**Table 2:** Demographic and clinical characteristics of the sample with impulsivity outcome

	*N* = 288
	*N* (%)
Male	146 (50.7)
Caucasian	232 (80.8)
Schizophrenia spectrum disorder	157 (54.5)
Bipolar spectrum disorder	131 (45.5)
Outpatient	207 (72.9)
Currently smoking	145 (50.9)
Use of statins	1 (0.3)

	Mean (*SD*)
BIS−11 total score	68.6 (10.8)
GAF‐S	53.5 (13.6)
GAF‐F	52.7 (13.6)
PANSS total score	52.2 (13.8)
TC in mmol/L	4.88 (0.95)
LDL‐C in mmol/L	3.06 (0.85)
HDL‐C in mmol/L	1.43 (0.44)

	Median (IQR)
Age	27 (13)
Years of education	12 (3)
PANSS‐EC	6 (3)
CDSS^a^	4 (7)
YMRS^a^	2 (4)
AUDIT^a^	5 (8)
DUDIT^a^	0 (4)
Antipsychotics^a^, ^b^	0.75 (1.50)
Lithium^a^, ^b^	0 (0)
Mood stabilizers^a^, ^b^	0 (0)
Antidepressants^a^, ^b^	0 (0.17)
BMI^a^	25.2 (6.2)

Abbreviations; AUDIT, Alcohol Use Disorders Identification Test; BIS‐11, Barratt Impulsiveness Scale; BMI, body mass index; CDSS, Calgary Depression Scale for Schizophrenia; DUDIT, Drug Use Disorders Identification Test; GAF‐F, Global Assessment of Functioning Split Version‐Function; GAF‐S, Global Assessment of Functioning Split Version‐Symptoms; HDL‐C, high‐density lipoprotein cholesterol; IQR, interquartile range; LDL‐C, low‐density lipoprotein cholesterol; PANSS, Positive and Negative Syndrome Scale; PANSS‐EC, Positive and Negative Syndrome Scale Excited Component; *SD*, standard deviation; TC, total cholesterol; YMRS, Young Mania Rating Scale.

^a^ Information about inpatient versus outpatient status available for *N* = 287, about smoking status for *N* = 285, about GAF‐F for *N* = 286, about PANSS total score for *N* = 285, about CDSS for *N* = 285, about YMRS for *N* = 234, about AUDIT for *N* = 283, about DUDIT for *N* = 286, about antipsychotics for *N* = 272, about lithium *N* = 276, about mood stabilizers *N* = 276, about antidepressants *N* = 275, and about BMI for *N* = 282.

^b^ Psychotropic medications as current daily dose relative to defined daily dose.

### 5.2 Aggression

**Figure 1:**
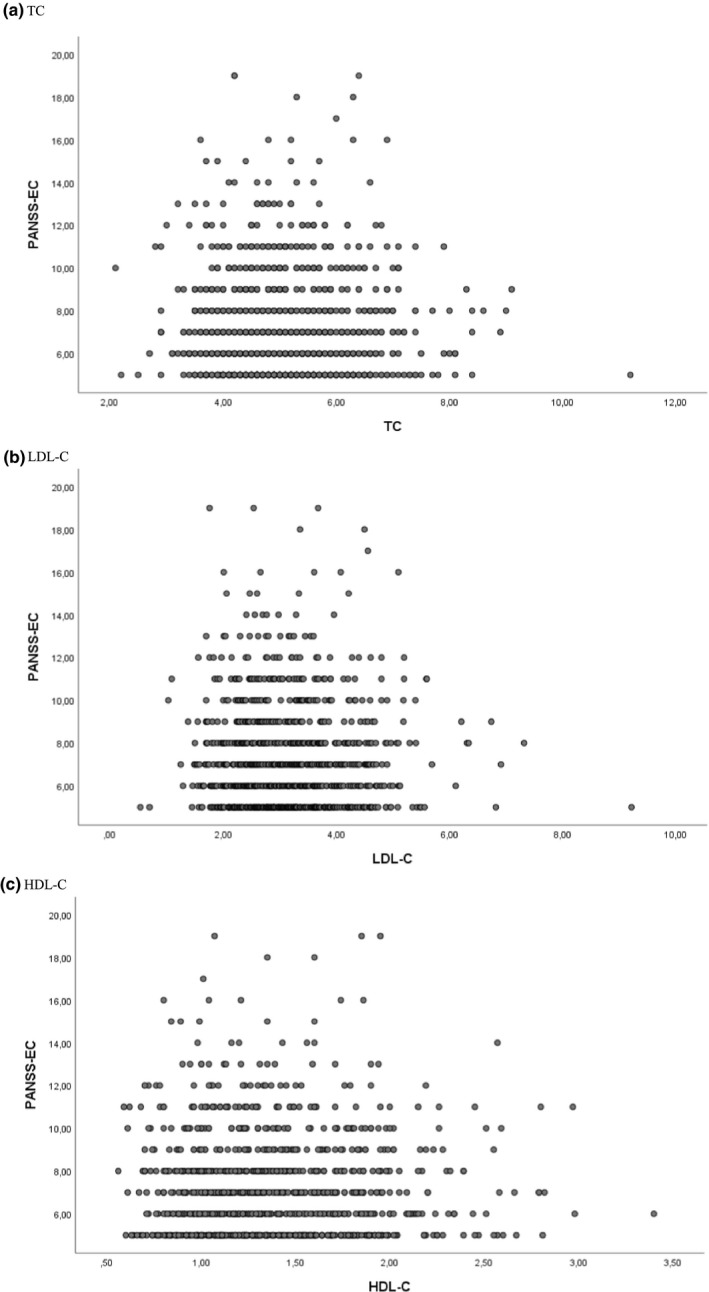
Aggression by cholesterol levels: (a) TC, (b) LDL‐C, (c) HDL‐C. Aggression measured by PANSS‐EC (y‐axis). Cholesterol levels in mmol/l (x‐axis). HDL‐C, high‐density lipoprotein cholesterol; LDL‐C, low‐density lipoprotein cholesterol; PANSS‐EC, Positive and Negative Syndrome Scale Excited Component; TC, total cholesterol

The raw data are presented in Figure 1. As shown in Table 3 and Table S1, there were no significant associations between TC, LDL‐C, or HDL‐C and aggression category when controlled for other covariates. There were no significant interactions between cholesterol levels (TC, LDL‐C, HDL‐C) and diagnostic group, inpatient versus outpatient status, or sex. Age was significantly negatively associated with aggression level (HLAS versus NAS (*p* < .001). There were no significant associations of sex, inpatient versus outpatient status, BMI, alcohol use, illicit substance use, or psychotropic medication with aggression category when controlled for other covariates. The total sample (*N* = 1 001) and the subsample with all covariates available (*N* = 689) yielded the same results, with two exceptions. Firstly, there was a significant association of diagnostic group with the HLAS versus NAS status in the total sample (*p* = .006), as opposed to no significant association of diagnostic group in the subsample. Secondly, the subsample showed an additional negative association of age with MLAS versus NAS status (*p* = .011).

**Table 3:** Multinomial logistic regression with aggression categories as dependent and TC as independent variable

	B (SE)	Wald	*p*	OR (95% CI)
HLAS versus NAS, total sample (*N* = 1001)				
TC	0.00 (0.12)	0.00	.971	1.00 (0.80 to 1.26)
Age	−0.05 (0.01)	13.37	**<.001**	0.95 (0.93 to 0.98)
Gender = male	−0.23 (0.23)	0.94	.332	0.80 (0.51 to 1.26)
Diagnosis = schizophrenia spectrum disorder^a^	0.70 (0.25)	7.69	**.006**	2.01 (1.23 to 3.29)
MLAS versus NAS, total sample (*N* = 1001)				
TC	−0.01 (0.07)	0.02	.895	0.99 (0.86 to 1.15)
Age	−0.01 (0.01)	3.00	.083	0.99 (0.97 to 1.00)
Gender = male	0.03 (0.15)	0.05	.823	1.03 (0.77 to 1.39)
Diagnosis = schizophrenia spectrum disorder^a^	0.11 (0.15)	0.55	.458	1.12 (0.83 to 1.51)
HLAS versus NAS, subsample (*N* = 689)				
TC	0.04 (0.16)	0.07	.796	1.04 (0.77 to 1.42)
Age	−0.05 (0.02)	9.52	**.002**	0.95 (0.92 to 0.98)
Gender = male	−0.33 (0.30)	1.22	.270	0.72 (0.40 to 1.30)
Diagnosis = schizophrenia spectrum disorder^a^	0.51 (0.35)	2.13	.144	1.67 (0.84 to 3.33)
Inpatient versus outpatient status = inpatient	−0.32 (0.34)	0.89	.346	0.73 (0.37 to 1.42)
BMI	−0.02 (0.03)	0.53	.467	0.98 (0.92 to 1.04)
AUDIT	0.03 (0.02)	1.79	.181	1.03 (0.99 to 1.08)
DUDIT	−0.00 (0.02)	0.04	.836	1.00 (0.96 to 1.04)
Antipsychotics	−0.05 (0.17)	0.10	.755	0.95 (0.68 to 1.32)
Antidepressants	−0.05 (0.19)	0.07	.798	0.95 (0.66 to 1.38)
Mood stabilizers	−0.15 (0.47)	0.10	.752	0.86 (0.35 to 2.15)
Lithium	−0.39 (0.53)	0.55	.459	0.68 (0.24 to 1.91)
MLAS versus NAS, subsample (*N* = 689)				
TC	0.02 (0.10)	0.03	.859	1.02 (0.84 to 1.24)
Age	−0.02 (0.01)	5.69	**.017**	0.98 (0.96 to 1.00)
Gender = male	0.08 (0.19)	0.17	.680	1.08 (0.75 to 1.57)
Diagnosis = schizophrenia spectrum disorder^a^	0.10 (0.22)	0.23	.630	1.11 (0.73 to 1.70)
Inpatient versus outpatient status = inpatient	−0.08 (0.22)	0.12	.730	0.93 (0.60 to 1.44)
BMI	−0.02 (0.02)	1.49	.223	0.98 (0.94 to 1.02)
AUDIT	−0.02 (0.02)	0.95	.329	0.99 (0.96 to 1.02)
DUDIT	−0.00 (0.02)	0.00	.955	1.00 (0.97 to 1.03)
Antipsychotics	−0.04 (0.11)	0.14	.711	0.96 (0.78 to 1.19)
Antidepressants	0.19 (0.11)	2.99	.084	1.21 (0.98 to 1.49)
Mood stabilizers	0.11 (0.25)	0.19	.660	1.12 (0.68 to 1.84)
Lithium	−0.32 (0.25)	1.71	.191	0.73 (0.45 to 1.17)

Abbreviations: AUDIT, Alcohol Use Disorders Identification Test; BMI, body mass index; CI, confidence interval; DUDIT, Drug Use Disorders Identification Test; HLAS group with higher levels of aggression symptoms; MLAS, group with minimal level of aggression symptoms; NAS, group with no aggression symptoms; OR, odds ratio; SE, standard error; TC, total cholesterol.

^a^ Diagnosis variable: schizophrenia spectrum disorder versus bipolar spectrum disorder.

*p* ≤ .017 in bold.

### 5.3 Impulsivity

**Figure 2:**
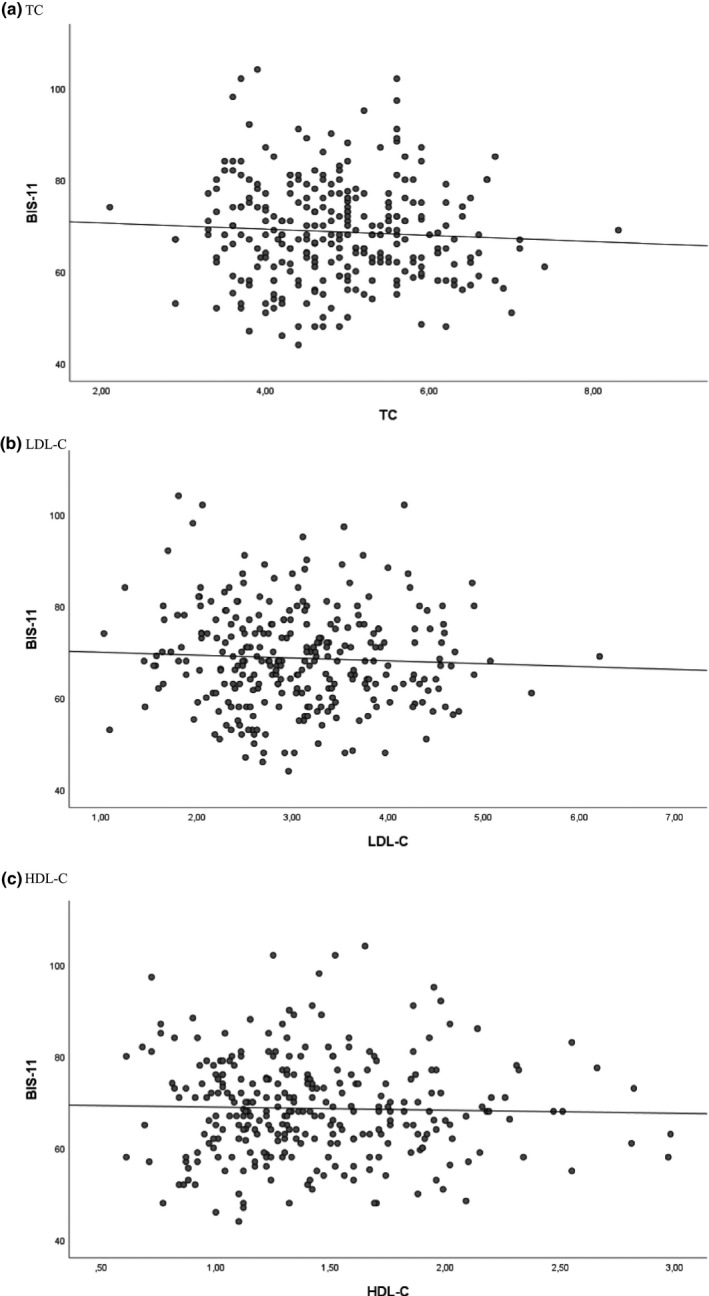
Scatterplot of cholesterol levels and impulsivity: (a) TC, (b) LDL‐C, (c) HDL‐C. Impulsivity measured by BIS‐11 total score (y‐axis). Cholesterol levels in mmol/l (x‐axis). Abbreviations: BIS‐11, Barratt Impulsiveness Scale; HDL‐C, high‐density lipoprotein cholesterol; LDL‐C, low‐density lipoprotein cholesterol; TC, total cholesterol

Scatterplots of cholesterol levels (TC, LDL‐C, HDL‐C) and impulsivity (BIS‐11 total score) are presented in Figure 2. As shown in Table 4 and Table S2, there were no significant associations between TC, LDL‐C, or HDL‐C and impulsivity when controlled for other covariates. There were no significant interactions between cholesterol levels (TC, LDL‐C, HDL‐C) and diagnostic group, inpatient versus outpatient status or sex. Illicit substance use was significantly positively associated with impulsivity (*p* < .001). There were no significant associations of sex, diagnosis, BMI, inpatient versus outpatient status, alcohol use, or use of psychotropic medication with impulsivity when controlled for other covariates. The total sample (*N* = 288) and the subsample with all covariates available (*N* = 259) yielded the same results, with exception of age being significantly negatively associated with impulsivity in the subsample (*p* = .016) as opposed to nonsignificant association in the total sample.

**Table 4:** Multiple linear regression with impulsivity scores as dependent and TC as independent variable

	B (SE)	β	*p*	95% CI for B
Total sample (*N* = 288)				
TC	−0.06 (0.72)	−0.01	.932	−1.48 to 1.36
Age	−0.15 (0.07)	−0.14	.025	−0.28 to −0.02
Gender^b^, ^a^	−0.13 (1.31)	−0.01	.924	−2.70 to 2.45
Diagnosis^a^, ^b^	−1.71 (1.33)	−0.08	.200	−4.33 to 0.91
Subsample (*N* = 259)				
TC	−0.30 (0.75)	−0.03	.688	−1.79 to 1.18
Age	−0.11 (0.07)	−0.10	.132	−0.25 to 0.03
Gender^b^, ^a^	0.99 (1.34)	0.05	.464	−1.66 to 3.63
Diagnosis^a^, ^b^	−2.20 (1.54)	−0.10	.154	−5.24 to 0.83
BMI	0.15 (0.14)	0.07	.263	−0.12 to 0.43
Inpatient versus outpatient status^b^, ^c^	−1.94 (1.63)	−0.08	.234	−5.15 to 1.26
AUDIT	0.22 (0.13)	0.12	.083	−0.03 to 0.47
DUDIT	0.41 (0.11)	0.26	**<.001**	0.19 to 0.62
Antipsychotics	−0.10 (0.70)	−0.01	.893	−1.48 to 1.29
Antidepressants	1.99 (0.99)	0.12	.045	0.04 to 3.93
Mood stabilizers	−1.38 (1.87)	−0.05	.463	−5.06 to 2.31
Lithium	−2.64 (2.03)	−0.08	.195	−6.64 to 1.36

Abbreviations: AUDIT, Alcohol Use Disorders Identification Test; B, unstandardized coefficient; BMI, body mass index; CI, confidence interval; DUDIT, Drug Use Disorders Identification Test; SE, standard error; TC, total cholesterol; β, standardized coefficient.

^a^ Gender variable: male = 0, female = 1.

^b^ Diagnosis variable: bipolar spectrum disorder = 0, schizophrenia spectrum disorder = 1.

^c^ Inpatient versus outpatient status: outpatient = 0, inpatient = 1.

*p* ≤ .017 in bold.

## 6 DISCUSSION

The main finding of this study was an absence of significant associations between cholesterol levels and aggression or impulsivity in a large naturalistic sample of patients with schizophrenia‐ and bipolar spectrum disorders. This is to date the largest clinical study of cholesterol and aggression or impulsivity in SMDs, expanding the scope of investigations to the outpatient psychiatric population. We found no significant interactions between cholesterol levels and diagnostic group or inpatient versus outpatient status on aggression or impulsivity.

In contrast to our hypothesis, lower cholesterol levels were not associated with higher levels of aggression or impulsivity. The literature on cholesterol and aggression in psychiatric populations is conflicting. Several studies have shown inverse associations between TC and aggression (Hillbrand et al., 1995; Mufti et al., 1998; Paavola et al., 2002; Roaldset et al., 2011; Suneson et al., 2019) or impulsivity (Kavoor et al., 2017; Troisi, 2011), whereas other studies have reported no significant associations between TC and aggression (Apter et al., 1999; Eriksen et al., 2017; Huang & Wu, 2000; Kavoor et al., 2017; Steinert et al., 1999) or impulsivity (Apter et al., 1999). Importantly, these were observational studies, and the findings may represent reverse causality with cholesterol levels reflecting the severity of distress among specific psychiatric populations. The negative findings in the current study are based on investigations among patients within a wide range of illness stages and severity, which may explain some of the discrepancy. As previously published (Gohar et al., 2019), an overlapping sample revealed no significant associations between TC and suicidal behavior. Considering the relationship between cholesterol levels and adverse mental symptoms in general, the meta‐analytic investigation of the relationship between TC and suicidality showing an inverse association is of importance (Wu et al., 2016). Since this meta‐analysis was based on cross‐sectional designs, causality remains unelucidated. Moreover, the need of exploring confounding variables has been highlighted in this context (Bartoli et al., 2017). Our results are more in line with results from RCTs in the general population which have not indicated adverse psychological effects of cholesterol lowering (Collins et al., 2016; Golomb et al., 2015; Muldoon et al., 2001). Experimental animal studies (Haagensen et al., 2014; Kaplan, Manuck, & Shively, 1991;) suggest that very high TC levels (above double the high end of the normal range) might indeed have protective effects against aggressive behavior. Interestingly, a negative association between prepubertal TC and adulthood impulsivity has been reported in healthy men (Tomson‐Johanson, Kaart, Kiivet, Veidebaum, & Harro, 2019). As aggression is a highly complex phenomenon, the possibility that unmeasured confounders overshadow weak associations between cholesterol levels and aggression cannot be ruled out. However, all lines of evidence taken together, findings do not suggest clinically significant adverse effects of cholesterol lowering on aggression in adults with SMDs.

Some studies, the present study included, aimed at estimating the association between exposure (i.e., cholesterol levels) and outcome (i.e., aggression), while other studies aspired prediction of the outcome using range of available covariates including variables such as history of violence or involuntary hospitalization (Roaldset et al., 2011). As such, complexity of the literature may be partly explained by these different methodological approaches motivated by different aims (Pearl, 2000). Despite extensive methodological heterogeneity, there are some common aspects of the previous research. Several previous studies have limited sample size (Kavoor et al., 2017; Mufti et al., 1998; Suneson et al., 2019). Many of the previous study designs involve testing for multiple cholesterol fractions (Eriksen et al., 2017; Kavoor et al., 2017; Paavola et al., 2002; Suneson et al., 2019; Troisi, 2011). However, none of these studies apply correction for multiple testing, implying a risk of false‐positive results. In terms of potential confounding, most of the previous study designs controlled for demographic variables such as sex and age, whereas the current study accounted also for diagnosis, medication, BMI, and substance use. These issues are addressed in the current study by the large sample size and stringent statistical approach. Moreover, a variety of aggression outcome measures are applied in the field. Findings linking low TC with aggressive behavior are based on studies that have operationalized registrations of aggressive behavior over a longer time period (Hillbrand et al., 1995; Mufti et al., 1998; Paavola et al., 2002; Roaldset et al., 2011), whereas studies that have used registrations acquired over a shorter time period (Apter et al., 1999; Huang & Wu, 2000; Kavoor et al., 2017; Steinert et al., 1999) do not show this link. This may suggest that trait properties of aggression implicated in this link can be descriptive of antisocial personality traits rather than of acts of aggression itself. This is supported by findings from criminal settings (Virkkunen, 1979) and general population (Freedman et al., 1995). Two outcome measures with different properties were used to assess aggression‐related behavior (Garcia‐Forero, Gallardo‐Pujol, Maydeu‐Olivares, & Andres‐Pueyo, 2009) in our study design. As such, aggression and impulsivity were addressed using both state (PANSS‐EC) and trait (BIS‐11) measures, encompassing both interviewer‐rated and questionnaire‐based approaches to outcome measure acquisition.

The main strengths of our study are representativeness of inpatient‐ and outpatient settings, thorough characterization of the sample, large sample size, and thus the opportunity of applying a stringent statistical approach. We controlled for potential confounding factors such as BMI, medication, and substance use, and we adjusted for multiple comparisons. These are methodological aspects targeting the key challenges in the field such as external validity and risk of false‐positive findings.

The present study also has some noteworthy limitations. Despite adjustments for a comprehensive range of variables, residual confounding cannot be ruled out. Moreover, as a consequence of the naturalistic study design, the highest levels of aggression were scarcely represented among the study participants. Exploratory plots presented in the current study did not indicate nonlinear associations between cholesterol and aggression or impulsivity; thus, no comprehensive investigations targeted at nonlinearity (Sedgwick, Young, Das, & Kumari, 2016) were conducted.

As of relevance for CVD risk management, the median age of patients in our sample was 29 years, which is below the recommended age of 40 years for initiation of systematic CVD risk evaluation and consecutive statin therapy initiation. As such, our sample with only 16 statin users was not suitable to investigate specific associations between statin use and adverse mental symptoms. Studies from psychiatric settings linking TC and aggression have been mainly conducted among statin nonusers (Mufti et al., 1998; Paavola et al., 2002; Suneson et al., 2019). There are neither findings from SMD populations (Leppien, Mulcahy, Demler, Trigoboff, & Opler, 2018) nor robust findings from the general population (Collins et al., 2016) supporting the concept of aggression as a side effect of statin use, independent of cholesterol levels.

## 7 CONCLUSION

We found no associations between cholesterol levels and aggression or impulsivity in this representative large sample of patients with schizophrenia‐ and bipolar spectrum disorders. This has clinical implications as CVDs are among leading causes of substantially reduced life expectancy in SMDs (Laursen et al., 2013), and patients with SMD are currently undertreated with statins (Mitchell, Lord, & Malone, 2012). An undertreatment of CVD risk in patients with comorbid SMD is a complex clinical challenge contributing to high morbidity and mortality rates in SMDs, implicating health care in both psychiatry and general practice (Jones, Howard, & Thornicroft, 2008; Leucht, Burkard, Henderson, Maj, & Sartorius, 2007; Woodhead et al., 2016).

## CONFLICT OF INTEREST

Ole Andreas Andreassen has received consultancy fees from HealthLytix and speaker's honorarium from Lundbeck. All other authors report no conflict of interest.

## AUTHOR CONTRIBUTION

GH, NES, and UKH designed the study with help from LMJ, RH, IM, OAA, and TVL. GH, NT, CB, SHL, LR, and MCFW participated in data collection and quality control. GH performed the statistical analyses. GH, LMJ, NES, and UKH wrote the first draft, and all authors critically revised and approved the manuscript.

### Peer Review

The peer review history for this article is available at https://publons.com/publon/10.1002/brb3.1751.

## Supporting information

Table S1‐S2

## Data Availability

The data are not publicly available due to privacy and ethical restrictions.
