# Supplementary material for: Disentangling the relationship between cholesterol, aggression, and impulsivity in severe mental disorders
Source: Brain Behav. 2020 Jul 17;10(9):e01751. doi: 10.1002/brb3.1751 (PMC7507477; doi:10.1002/brb3.1751)
Supplement: Supplementary file 1 — Table S1‐S2 [file BRB3-10-e01751-s001.docx]

Table S1 a). Multinomial logistic regression with aggression categories as dependent and LDL-C as independent variable

|  | B (SE) | Wald | p | OR (95% CI) |
| --- | --- | --- | --- | --- |
| HLAS versus NAS, total sample (N = 1 001) | | |  |  |
| LDL-C | 0.03 (0.13) | 0.04 | 0.844 | 1.03 (0.80 to 1.32) |
| Age | -0.05 (0.01) | 14.08 | **p < 0.001** | 0.95 (0.93 to 0.98) |
| Gender = male | -0.23 (0.24) | 0.98 | 0.322 | 0.79 (0.50 to 1.26) |
| Diagnosis = schizophrenia spectrum disorder^†^ | 0.69 (0.25) | 7.56 | **0.006** | 2.00 (1.22 to 3.28) |
| MLAS versus NAS, total sample (N = 1 001) | | | | |
| LDL-C | -0.01 (0.08) | 0.00 | 0.954 | 1.00 (0.85 to 1.17) |
| Age | -0.01 (0.01) | 3.21 | 0.073 | 0.99 (0.97 to 1.00) |
| Gender = male | 0.03 (0.15) | 0.05 | 0.823 | 1.03 (0.77 to 1.39) |
| Diagnosis = schizophrenia spectrum disorder^†^ | 0.11 (0.15) | 0.54 | 0.463 | 1.12 (0.83 to1.51) |
| HLAS versus NAS, subsample (N = 689) | | | | |
| LDL-C | 0.11 (0.18) | 0.36 | 0.546 | 1.11 (0.79 to 1.57) |
| Age | -0.06 (0.02) | 10.11 | **0.001** | 0.95 (0.91 to 0.98) |
| Gender = male | -0.36 (0.31) | 1.37 | 0.241 | 0.70 (0.38 to 1.27) |
| Diagnosis = schizophrenia spectrum disorder^†^ | 0.50 (0.35) | 2.05 | 0.152 | 1.66 (0.83 to 3.30) |
| Inpatient versus outpatient status = inpatient | -0.32 (0.34) | 0.87 | 0.352 | 0.73 (0.37 to 1.42) |
| BMI | -0.03 (0.03) | 1.63 | 0.427 | 0.98 (0.92to 1.04) |
| AUDIT | 0.03 (0.02) | 1.77 | 0.183 | 1.03 (0.99 to 1.08) |
| DUDIT | -0.01 (0.02) | 0.05 | 0.820 | 1.00 (0.96 to 1.04) |
| Antipsychotics | -0.06 (0.17) | 0.11 | 0.746 | 0.95 (0.68 to 1.32) |
| Antidepressants | -0.05 (0.19) | 0.08 | 0.776 | 0.95 (0.65 to 1.38) |
| Mood stabilizers | -0.14 (0.47) | 0.09 | 0.769 | 0.87 (0.35 to 2.18) |
| Lithium | -0.38 (0.53) | 0.52 | 0.473 | 0.68 (0.38 to 1.27) |
| MLAS versus NAS, subsample (N = 689) | | | | |
| LDL-C | 0.06 (0.11) | 0.25 | 0.616 | 1.06 (0.85 to 1.32) |
| Age | -0.02 (0.01) | 6.22 | **0.013** | 0.98 (0.96 to 1.00) |
| Gender = male | 0.07 (0.19) | 0.12 | 0.733 | 1.07 (0.74 to 1.55) |
| Diagnosis = schizophrenia spectrum disorder^†^ | 0.10 (0.22) | 0.20 | 0.652 | 1.10 (0.72 to 1.68) |
| Inpatient versus outpatient status = inpatient | -0.08 (0.23) | 0.11 | 0.737 | 0.93 (0.60 to 1.44) |
| BMI | -0.03 (0.02) | 1.62 | 0.203 | 0.98 (0.94 to 1.01) |
| AUDIT | -0.02 (0.02) | 0.96 | 0.327 | 0.99 (0.96 to 1.02) |
| DUDIT | -0.00 (0.02) | 0.01 | 0.942 | 1.00 (0.97 to 1.03) |
| Antipsychotics | -0.04 (0.11) | 0.14 | 0.706 | 0.96 (0.78 to 1.19) |
| Antidepressants | 0.19 (0.11) | 2.94 | 0.086 | 1.20 (0.97 to 1.49) |
| Mood stabilizers | 0.12 (0.25) | 0.22 | 0.643 | 1.13 (0.68 to 1.85) |
| Lithium | -0.32 (0.24) | 1.70 | 0.193 | 0.73 (0.45 to 1.17) |

Table S1 b). Multinomial logistic regression with aggression categories as dependent and HDL-C as independent variable

|  | B (SE) | Wald | p | OR (95% CI) |
| --- | --- | --- | --- | --- |
| HLAS versus NAS, total sample (N = 1 001) | | |  |  |
| HDL-C | -0.40 (0.31) | 1.69 | 0.194 | 0.67 (0.37 to 1.23) |
| Age | -0.05 (0.01) | 14.76 | **p < 0.001** | 0.95 (0.93 to 0.98) |
| Gender = male | -0.34 (0.25) | 1.90 | 0.168 | 0.71 (0.44 to 1.16) |
| Diagnosis = schizophrenia spectrum disorder^†^ | 0.67 (0.25) | 7.02 | **0.008** | 1.95 (1.19 to 3.19) |
| MLAS versus NAS, total sample (N = 1 001) | | | | |
| HDL-C | -0.03 (0.19) | 0.03 | 0.860 | 0.97 (0.67 to 1.40) |
| Age | -0.01 (0.01) | 3.66 | 0.056 | 0.99 (0.98 to 1.00) |
| Gender = male | 0.02 (0.16) | 0.02 | 0.894 | 1.02 (0.75 to 1.40) |
| Diagnosis = schizophrenia spectrum disorder^†^ | 1.11 (0.15) | 0.51 | 0.475 | 1.11 (0.83 to1.50) |
| HLAS versus NAS, subsample (N = 689) | | | | |
| HDL-C | -0.64 (0.41) | 2.48 | 0.116 | 0.53 (0.24 to 1.17) |
| Age | -0.05 (0.02) | 9.07 | **0.003** | 0.95 (0.92 to 0.98) |
| Gender = male | -0.52 (0.32) | 2.56 | 0.110 | 0.60 (0.32 to 1.12) |
| Diagnosis = schizophrenia spectrum disorder^†^ | 0.53 (0.35) | 2.24 | 0.135 | 1.69 (0.85 to 3.37) |
| Inpatient versus outpatient status = inpatient | -0.30 (0.34) | 0.76 | 0.384 | 0.74 (0.38 to 1.45) |
| BMI | -0.04 (0.03) | 1.40 | 0.237 | 0.96 (0.90 to 1.03) |
| AUDIT | 0.03 (0.02) | 2.03 | 0.154 | 1.03 (0.99 to 1.08) |
| DUDIT | -0.01 (0.02) | 1.11 | 0.745 | 0.99 (0.95 to 1.04) |
| Antipsychotics | -0.04 (0.17) | 0.07 | 0.799 | 0.95 (0.66 to 1.38) |
| Antidepressants | -0.05 (0.19) | 0.06 | 0.806 | 0.96 (0.66 to 1.38) |
| Mood stabilizers | -0.09 (0.47) | 0.04 | 0.851 | 0.92 (0.36 to 2.30) |
| Lithium | -0.39 (0.53) | 0.56 | 0.454 | 0.68 (0.24 to 1.89) |
| MLAS versus NAS, subsample (N = 689) | | | | |
| HDL-C | -0.27 (0.24) | 1.21 | 0.271 | 0.77 (0.48 to 1.23) |
| Age | -0.02 (0.01) | 5.56 | 0.018 | 0.98 (0.96 to 1.00) |
| Gender = male | -0.01 (0.20) | 0.00 | 0.980 | 1.00 (0.67 to 1.48) |
| Diagnosis = schizophrenia spectrum disorder^†^ | 0.11 (0.22) | 0.27 | 0.605 | 1.12 (0.73 to 1.71) |
| Inpatient versus outpatient status = inpatient | -0.07 (0.23) | 0.09 | 0.765 | 0.94 (0.60 to 1.45) |
| BMI | -0.03 (0.02) | 2.26 | 0.133 | 0.97 (0.93 to 1.01) |
| AUDIT | -0.01 (0.02) | 0.83 | 0.362 | 0.99 (0.96 to 1.02) |
| DUDIT | -0.00 (0.02) | 0.02 | 0.896 | 1.00 (0.97 to 1.03) |
| Antipsychotics | -0.04 (0.11) | 0.10 | 0.747 | 0.97 (0.78 to 1.20) |
| Antidepressants | 0.19 (0.11) | 3.07 | 0.080 | 1.21 (0.98 to 1.49) |
| Mood stabilizers | 0.14 (0.25) | 0.29 | 0.588 | 1.15 (0.70 to 1.89) |
| Lithium | -0.33 (0.25) | 1.76 | 0.185 | 0.72 (0.45 to 1.17) |

^†^ Diagnosis variable: schizophrenia spectrum disorder versus bipolar spectrum disorder

Abbreviations: AUDIT, Alcohol Use Disorders Identification Test; BMI, Body mass index; CI, confidence interval; DUDIT, Drug Use Disorders Identification Test; HDL-C, high-density lipoprotein cholesterol; HLAS group with higher levels of aggressive symptoms; LDL-C, low-density lipoprotein cholesterol; MLAS, group with minimal level of aggressive symptoms; NAS, group with no aggressive symptoms; OR, odds ratio; SE, standard error.

| Table S2 a). Multiple linear regression with impulsivity scores as dependent and LDL-C as independent variable | | | | |
| --- | --- | --- | --- | --- |
|  | B (SE) | β | p | 95% CI for B |
| Total sample (N = 288) | | | | |
| LDL-C | -0.04 (0.81) | -0.00 | 0.963 | -1.64 to 1.56 |
| Age | -0.15 (0.06) | -0.14 | 0.022 | -0.28 to -0.02 |
| Gender^†^ | -0.13 (1.35) | -0.006 | 0.924 | -2.78 to 2.52 |
| Diagnosis^‡^ | -1.72 (1.33) | -0.08 | 0.196 | -4.33 to 0.89 |
| Subsample (N = 259) |  |  |  |  |
| LDL-C | -0.27 (0.86) | -0.02 | 0.754 | -1.96 to 1.42 |
| Age | -0.11 (0.07) | -0.11 | 0.109 | -0.25 to 0.03 |
| Gender^†^ | 0.94 (1.37) | 0.04 | 0.494 | -1.76 to 3.63 |
| Diagnosis^‡^ | -2.24 (1.53) | -0.10 | 0.146 | -5.26 to 0.79 |
| BMI | 0.16 (0.14) | 0.07 | 0.259 | -0.12 to 0.43 |
| Inpatient versus outpatient status^§^ | -1.95 (1.63) | -0.08 | 0.232 | -5.16 to 1.26 |
| AUDIT | 0.22 (0.13) | 0.12 | 0.085 | -0.03 to 0.47 |
| DUDIT | 0.41 (0.11) | 0.26 | **p < 0.001** | 0.19 to 0.62 |
| Antipsychotics | -0.10 (0.70) | -0.01 | 0.885 | -1.49 to 1.29 |
| Antidepressants | 1.97 (0.99) | 0.12 | 0.047 | 0.03 to 3.92 |
| Mood stabilizers | -1.36 (1.87) | -0.05 | 0.468 | -5.05 to 2.33 |
| Lithium | -2.66 (2.03) | -0.08 | 0.191 | -6.66 to 1.33 |

| Table S2 b). Multiple linear regression with impulsivity scores as dependent and HDL-C as independent variable | | | | |
| --- | --- | --- | --- | --- |
|  | B (SE) | β | p | 95% CI for B |
| Total sample (N = 288) | | | | |
| HDL-C | -0.60 (1.59) | -0.02 | 0.708 | -3.73 to 2.54 |
| Age | -0.15 (0.06) | -0.14 | 0.018 | -0.27 to -0.03 |
| Gender^†^ | 0.10 (1.42) | 0.01 | 0.944 | -2.69 to 2.89 |
| Diagnosis^‡^ | -1.73 (1.31) | -0.08 | 0.186 | -4.31 to 0.95 |
| Subsample (N = 259) |  |  |  |  |
| HDL-C | -0.25 (1.69) | -0.01 | 0.883 | -3.58 to 3.08 |
| Age | -0.12 (0.07) | -0.11 | 0.090 | -0.25 to 0.02 |
| Gender^†^ | 1.09 (1.43) | 0.05 | 0.446 | -1.73 to 3.92 |
| Diagnosis^‡^ | -2.29 (1.52) | -0.11 | 0.134 | -5.29 to 0.71 |
| BMI | 0.14 (0.15) | 0.07 | 0.326 | -0.14 to 0.43 |
| Inpatient versus outpatient status^§^ | -1.98 (1.63) | -0.08 | 0.226 | -5.18 to 1.23 |
| AUDIT | 0.22 (0.13) | 0.12 | 0.087 | -0.03 to 0.47 |
| DUDIT | 0.40 (0.11) | 0.26 | **p < 0.001** | 0.19 to 0.62 |
| Antipsychotics | -0.09 (0.71) | -0.01 | 0.895 | -1.48 to 1.29 |
| Antidepressants | 1.99 (0.99) | 0.12 | 0.045 | 0.04 to 3.93 |
| Mood stabilizers | -1.35 (1.87) | -0.05 | 0.471 | -5.04 to 2.34 |
| Lithium | -2.62 (2.03) | -0.08 | 0.198 | -6.62 to 1.38 |

^†^Gender variable: Male = 0, Female = 1

^‡^ Diagnosis variable: Bipolar spectrum disorder = 0, Schizophrenia spectrum disorder = 1

^§^ Inpatient versus outpatient status: Outpatient = 0, Inpatient = 1

Abbreviations: AUDIT, Alcohol Use Disorders Identification Test; B, unstandardized coefficient; BMI, Body mass index; CI, confidence interval; DUDIT, Drug Use Disorders Identification Test; HDL-C, high-density lipoprotein cholesterol; LDL-C, low-density lipoprotein cholesterol; SE, standard error; β, standardized coefficient.
